# Comparing the effects of olfactory-based sensory stimulation and board game training on cognition, emotion, and blood biomarkers among individuals with dementia: A pilot randomized controlled trial

**DOI:** 10.3389/fpsyg.2022.1003325

**Published:** 2022-09-20

**Authors:** Li-jung Lin, Kuan-yi Li

**Affiliations:** ^1^Graduate Institute of Sport, Leisure, and Hospitality Management, National Taiwan Normal University, Taipei, Taiwan; ^2^Department of Occupational Therapy, Graduate Institute of Behavioral Sciences, College of Medicine, Chang Gung University, Taoyuan, Taiwan; ^3^Movement Disorders Section, Department of Neurology, Linkou Chang Gung Memorial Hospital, Taoyuan, Taiwan; ^4^Healthy Aging Research Center, Chang Gung University, Taoyuan, Taiwan

**Keywords:** olfactory dysfunction, multisensory stimulation, board game, Alzheimer’s disease, immunomagnetic reduction, Tau, cognition, depression

## Abstract

Olfactory dysfunction can indicate early cognitive decline and is associated with dementia symptoms. We developed an olfactory-based sensory stimulation program and investigated its effects on cognition and emotion, and board game training were used as a comparison. In this parallel design pilot study, 30 participants with mild to moderate dementia were equal randomly assigned to the control (CONT), olfactory stimulation with cognitive training (OS), and board game (BG) groups. Two participants were withdrawn from CONT and OS groups, respectively. The intervention was a 12-week program with one 30-min session twice a week. We employed a blood-based biomarker technique and several cognitive and psychological tests to measure basal and after-intervention values. No significant differences were observed between the groups after intervention, as measured using the Mini-Mental State Examination, Loewenstein Occupational Therapy Cognitive Assessment (LOTCA), Top International Biotech Smell Identification Test, and Geriatric Depression Scale (GDS). The results showed that the OS group had a lower plasma Tau level than the other groups following intervention, whereas the CONT group had a significantly increased plasma amyloid ß1-42 level. OS participants had a lower concentration ratio of plasma Tau and amyloid Aß1-42 and showed more stable or improved cognition, olfactory function, and mood state. Both the OS and BG groups had a higher percentage of participants with stable or improved cognition and emotion. Taken together, these results suggest that olfactory-based sensory stimulation can be a beneficial intervention for patients with dementia.

**Clinical trial registration:**

[Clinicaltrials.gov], identifier [NCT05168098].

## Introduction

Dementia, a general term for degenerative brain diseases, is characterized by a decline in thinking (i.e., calculation, judgment, and abstract thinking) and memory, accompanied by emotional and language difficulties. Neuropsychiatric symptoms are as important as cognitive symptoms, resulting in the need for full day care in the later stages and representing a burden of care for family and professional caregivers (Cerejeira et al., 2012; Seidel and Thyrian, 2019). Almost 90% of people with dementia may experience various behavioral and psychological symptoms, including depression, anxiety, irritability, aggression, apathy, sleeping disturbance, and wandering (Strøm et al., 2016; Dimitriou and Tsolaki, 2017).

Compared with pharmacotherapy, non-pharmacological interventions have been recognized as having fewer adverse effects and being a cost-effective approach for improving patients’ quality of life (Pinto et al., 2020). Several well-known treatments and therapies have been proposed, such as cognitive-emotional interventions, sensory stimulation interventions, behavior management techniques, and physical exercise (Dimitriou and Tsolaki, 2017). In particular, sensory stimulation is widely used for people with dementia, especially those living in long-term care facilities, because they may have either sensory deprivation or too much sensory stimulation (Milev et al., 2008).

Sensory stimulation interventions usually use direct sensory stimulation activities to engage perception or passively immerse patients in their surroundings to evoke their five senses (e.g., vision, hearing, smell, taste, and touch (Witucki and Twibell, 1997). A range of activities can be implemented using different types of stimuli, including music, gardens, light, massage or acupressure, therapy animals, odors or aromas, and the environment. Some interventions rely on single-sense strategies (unisensory stimulation, e.g., music therapy, light therapy, and aromatherapy), whereas others harness several senses (multisensory stimulation, e.g., Snolezen, Sonas). Existing mild-to-moderate quality of evidence supports occupation- and environment-based multisensory activities (i.e., light, gardening, music, Yago, and aromatherapy) for patients with dementia (Smith and D’Amico, 2020), primarily to reduce depression, anxiety, cognition, and agitation symptoms (Strøm et al., 2016).

Sensory stimulation interventions mainly target patients with moderate-to-severe dementia, because reduced intellectual reasoning, limited verbal skills, and restless or agitated behavior may result in difficulty attending traditional therapy or leisure activities (Spaull et al., 1998). However, these interventions may also slow down deterioration and increase daily autonomy in patients with less severe dementia (Pinto et al., 2020). Baker et al. have found that adults with dementia can benefit from multisensory stimulation or traditional activities, with improvement in both their emotional and social responses (Baker et al., 2001, 2003). Most studies on sensory stimulation have focused on neuropsychiatric effects; only a few have examined the impact of interventions on sensory perception and cognition (Pinto et al., 2020).

Interestingly, smell dysfunction can indicate dementia onset and could be an early biomarker of cognitive decline (Devanand et al., 2015). The presence of senile plaques and neurofibrillary tangles in the olfactory bulb has been associated with cognition deterioration among patients diagnosed with dementia, particularly Alzheimer’s disease (Christen-Zaech et al., 2003).

Several olfactory tests have been developed to detect the onset of early-stage cognitive impairment (Sun et al., 2012). However, to the best of our knowledge, no study has used an olfactory test to examine the effects of interventions on the perception of olfactory function. Some studies have demonstrated that odor exposure can effectively evoke autobiographical memories and trigger strong feelings about a previous event, especially for individuals with mild Alzheimer’s disease or related dementia (El Haj et al., 2017; Glachet et al., 2019; Pinto et al., 2020). Although these researchers have suggested that olfactory stimulation can assist with memory retrieval, its effects on cognition improvement and the delay of dementia progression have not been verified. Nevertheless, cognitive training involving sensory stimulation and repetitive cognitive tasks could promote synaptic growth and neurophysiological cognitive changes among people with mild-to-moderate dementia (Martin-Lopez et al., 2021). Non-verbal or -visuospatial stimuli are commonly used during such tasks (Miotto et al., 2018), but olfactory stimuli are limited. Recently, most studies have only examined the intervention effect of sensory stimulation or cognitive training alone. Instead, a study has tested the combination of cognitive training and olfactory sensory stimulation. Based on the assumption of olfactory stimulation and memory retrieval, we would like to propose an innovative approach by combining the olfactory stimuli and cognitive training to testify if it is an effective intervention for dementia.

Detection of cognitive decline is often difficult, making direct cognitive measurement an ill-suited approach for verifying the effectiveness of interventions (Butler et al., 2018). However, immunomagnetic reduction (IMR) can be used as an alternative approach to calculate the degree of cognitive decline in patients with mild cognitive impairment (Tang et al., 2021). Thus, a novel blood-based biomarker technique harnessing IMR has been developed to quantify target molecules such as amyloid ß1-42 (Aß1-42) and the tau protein (Tau) using a magnetic reagent (Chiu et al., 2013; Yang et al., 2017). As for plasma Aß1-42, depending on the assay methodology, plasma concentrations have been found to increase or decrease in patients with dementia compared to normal controls (Udeh-Momoh et al., 2022). Both Aß1-42 and Tau have been found at abnormal levels in patients with dementia. Thus, plasma Tau levels are elevated in patients with Alzheimer’s disease, frontotemporal disease, or vascular dementia compared to normal controls (Mattsson et al., 2016; Rost et al., 2022). Further, it could indicate a natural cognitive decline due to disease progression. In contrast, plasma Tau levels increase with brain atrophy (Chiu et al., 2014; Fan et al., 2018). Hence, Aß1-42 and Tau plasma levels as measured through IMR increase with the severity of cognitive impairment. Therefore, the use of this highly sensitive and specific test to detect changes in plasma markers allows for examining the effectiveness of interventions for Alzheimer’s disease and related dementia. However, we might be the first research to explore the non-pharmacological intervention effects among people with dementia. Nonetheless, there is one research to test the effect of traditional herbal medicine in an Alzheimer’s disease model on the animal trial (Su et al., 2022).

Therefore, the purpose of this study was to examine the effects of an olfactory-based sensory stimulation protocol comparing with the board game training in older adults with mild-to-moderate dementia in a day care center. We used IMR, cognitive tests, and psychological scales to measure multiple patient outcomes.

## Materials and methods

### Participant recruitment

We performed a single-blinded, randomized controlled trial with a parallel design to compare the effects of olfactory-based sensory stimulation activities (olfactory simulation group, OS) with two credible control conditions consisting of board game activities (board game group, BG) and regular routine activities (control group, CONT). Two day care centers for older persons in Taipei City participated in the study; the centers’ staff assisted with the recruitment of qualified participants and obtainment of informed consent from family members. The research protocol was approved by the Research Ethics Committee of the National Taiwan Normal University (NTNU-REC 201901HM030). The protocol was also registered with Clinical trials. gov, identifier: NCT05168098.

Inclusion criteria for the participants included being aged >50 years and having a physician-assigned diagnosis of mild or moderate dementia (Clinical Dementia Rating = 1–2); being admitted to the daycare center and living in the community; and being willing to undergo blood tests with a family member’s consent. The exclusion criteria included having a history of chronic rhinitis or sinusitis, which may cause loss of smell; being unable to perform the cognitive exam or olfactory test (e.g., having a severe hearing impairment); or being unable to attend group activities due to severe emotional or aggressive behaviors.

**FIGURE 1:**
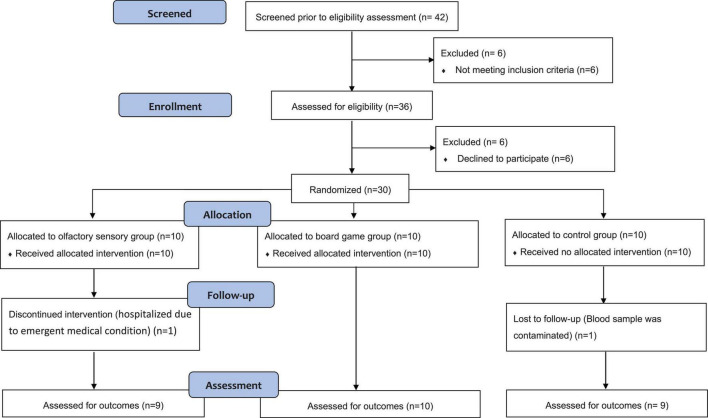
CONSORT flow diagram for the pilot and feasibility trial.

Olfactory-based stimulation therapy is a novel intervention approach; therefore, the adequate sample size could not be calculated by power calculation. Based on previous pilot studies in patients with dementia using multisensory stimulation (Witucki and Twibell, 1997; Milev et al., 2008; Maseda et al., 2014; Clements-Cortes et al., 2016), we planned to recruit 10 participants for each group. The first author conducted the allocation concealment and assigned the participants to the group directly. A total of 30 participants were recruited from 2 units (nine from unit A and 21 from unit B) and generated three comparison groups using computerized block randomization, with an equal allocation ratio, by referring to a table of random numbers. However, two participants withdrew mid-study due to emergent medical conditions (from unit B), and one blood sample was contaminated (from unit A). Figure 1 shows the flow chart of participant involvement throughout the research period. In total, 28 participants (8 men, 20 women) completed the study (see Table 1), including 9 participants in the OS (2 men, 7 women), 10 participants in the BG (4 men, 6 women), and 9 participants in the CONT (2 men, 7 women) groups. The participants’ average age was 82 years (range 67-92 years).

**TABLE 1:** Demographical background of participants.

	Olfactory group		Board game group		Control group		Total	
Age (M ± SD)	78 ± 6.4		80.2 ± 8.1		87.9 ± 2.8		81.96 ± 7.4	

**Sex**	***n***	**%**	***n***	**%**	***n***	**%**	***n***	**%**

Man	2	22.2	4	40	2	22.3	8	28.6
Woman	7	77.8	6	60	7	77.8	20	71.4
**Educational level**								
None	0	0	1	10	1	11.1	2	7.1
Elementary	4	44.4	5	50	3	33.3	12	42.9
High school	5	55.6	4	40	5	55.6	14	50
**Unit**								
A	3	33.3	3	30	2	22.2	8	28.6
B	6	66.7	7	70	7	77.8	20	71.4

### Interventions and procedures

The BG and OS groups participated in a 30-min intervention session twice a week for 3 months (total, 12 weeks). Both the BG and OS group participated in a series of small-group (3-5 persons) sessions, respectively, in which cognitive training or olfactory sensory stimulation tasks were performed. The interventions were conducted between January 2 and March 30, 2020, in A unit and between July 17 and September 21, 2020, in B unit. Two group activities were delivered on the same day but during different sessions.

In the OS group, 15 flavors of essential oils (i.e., lavender, rosemary, sweet orange, lemongrass, mint, and hinoki) and essences (i.e., lemon, coffee, peach, magnolia, chocolate, jasmine, strawberry, pomelo, and passion fruit) were used, with two to three flavors purposefully selected for each session. Because some flavors were familiar to the participants and others were not, the familiar flavors were initially used to trigger memory. The unfamiliar flavors (i.e., lavender, rosemary, sweet orange, and lemon) previously recognized as having potential effects on cognitive function were used in later sessions (Jimbo et al., 2009).

To enhance odor discrimination and identification ability, the participants were asked to smell the flavor from a composition cork and find the matching picture. The olfactory stimulation intervention consisted in a cognitive training approach to perform a set of cognitive tasks with an increasing level of difficulty. During the session, within 30 min, 3–5 flavors were used as the principal target, and the instructor will provide a hint with verbal or visual cues to assist the participant in guessing the name of the flavor. The research team handmade the game kits, including the smell corks and pictures. While several board game processes were effectively harnessed during the OS intervention, including memory, dice rolling, set collecting, and storytelling, to allow comparison with the OS intervention the BG activities were focused on visual, hearing, and tactile stimuli rather than smelling. Notably, board game involving multiple cognitive tasks, such as memory, attention, or executive functions (Martin-Lopez et al., 2021), has recently been popular in the community and have shown benefits in global cognition and executive function for older adults (Chen et al., 2022). We adopted the board game as a comparison to testify if olfactory stimulation is an improved effective intervention for dementia.

The BG group was exposed to 24 board games commonly used among the elderly population in Taiwan, including Noah’s Ark, Splash Attack, Pengoloo, Speedy, and Zingo. Because some of the games were too complex for the participants to play, selected rules were modified toward more simple and directed thinking. To replicate the cognitive training approach in the OS group, the board games in the BG group were selected to involve the same game mechanisms. Activities in both the OS and BG groups were delivered by an instructor who was a research assistant trained by a certified recreational therapist.

The CONT group did not participate in any of the aforementioned activities but continued following the center’s daily routine, including exercise, recreational activities, arts and crafts, cognitive training activities, and health education. Both the OS and BG groups also attended these activities.

### Assessment instruments

Cognitive abilities were assessed in all participants before (Week 0) and after (Week 12) the interventions with blood tests, olfactory tests, cognitive examinations, and psychological measurements. One research assistant as an independent rater was trained and blinded following the group assignment procedure to perform the cognitive, olfactory, and psychological measurements.

#### Immunomagnetic reduction blood tests

A registered nurse drew 5 ml of blood from each participant twice at baseline and immediately after the intervention. The laboratory staff analyzing the samples was blinded to the group assignment.

##### Stage I: Plasma preparation

K3 EDTA tubes (10 ml; 455036, Greiner) were used to collect the blood samples, with the subsequent immediate gentle inversion of each tube 10×. The tubes were centrifuged at room temperature at 2,500 *g* for 15 min using a bracket rotor. Every 1 ml of plasma (supernatant) was transferred to a fresh 1.5-ml Eppendorf tube using a disposable 1-ml micropipette tip. All the samples were frozen at −80°C before measurements.

##### Stage II: Plasma biomarker assays

IMR kits were used to assay the Aß1-42 (MF-AB2-0060; MagQu) and Tau (MF-TAU-0060; MagQu) biomarkers. For Aß1-42, 60 μl of reagent was mixed with 60 μl of plasma. For Tau, 80 μl of reagent was mixed with 40 μl of plasma. Duplicated measurements were performed for each biomarker, and their average value was reported. An IMR analyzer (XacPro-S; MagQu) was used to detect the samples’ biomarker concentrations. For each measurement batch, calibrators (CA-DEX-0060 and CA-DEX-0080; MagQu) and control solutions (CL-AB2-000T, CL-AB2-020T, CL-TAU-000T, and CL-TAU-050T; MagQu) were used.

#### Cognitive examinations

Two common cognitive examinations, namely the MMSE and the Loewenstein Occupational Therapy Cognitive Assessment-Geriatric (LOTCA-G), were performed to detect changes before and after intervention. The MMSE is a widely used tool for clinical evaluation, with five major domains (orientation, registration, attention and calculation, recall, and language). The examination relies on 11 questions; the total scores range from 0 to 30, with a higher score indicating better cognitive function (Folstein et al., 1975). The cut-off score of 23 has been suggested for detecting dementia when using the MMSE (Kochhann et al., 2010).

The LOTCA-G, a modified version of the LOTCA for adults aged >70 years or for clients with a slower response to cognitive tasks, contains 23 subsets in seven cognitive areas (orientation, visual perception, spatial perception, praxis, visuomotor organization, thinking operations, and memory). Each question is scored from 1 (severe deficit) to 4 (average performance), with total scores ranging from 23 to 100 and higher scores indicating better cognitive performance (Bar-Haim Erez and Katz, 2004). LOTCA-G, a well-established test of discriminant validity for individuals with dementia, can be used to monitor changes in cognitive function during an intervention.

#### Olfactory test

The Top International Biotech Smell Identification Test (TIBSIT) was used to assess olfactory function. TIBSIT, a commercialized test, is a new version of the Taiwan Smell Identification Test that uses a scratch-and-sniff format rather than liquid-jar odorants (Hsieh et al., 2021). TIBSIT, which consists of a questionnaire with 16 tests (two repeated tests for each odor), uses eight odors relevant to the Taiwanese population. Each participant completed the test with the assistance of a master’s student, if necessary.

During a test, the participant scratches fragrant microcapsules off a piece of paper using a pencil, smells the fragrance, and then answers two questions. The first question offers a single choice to identify the closest odor among four names, and the second question offers a three-item choice of not detectable (smells nothing, 0 points); detectable, but not sure (can smell something but unsure, 1 point); and detectable (can smell and know exactly what it is, 2 points). Thus, if one selects the correct odor name with the “detectable” answer, one receives the maximum score of 3 points for a test. If one selects the “detectable, but not sure” answer, one receives only 2 points, and if one selects the wrong answer with “detectable, but not sure,” one still receives 1 point; however, if one selects the wrong answer with “detectable,” one would receive 0 points. A total of 48 points are available for 16 tests.

#### Psychological measurement

We used the Geriatric Depression Scale (GDS-15), an easy-to-conduct 15-item questionnaire, to assess participants’ emotional changes during the intervention. Answers are reported using a yes-no scale; the total scores range from 0 to 15, with higher scores indicating more severe depression (Sheikh and Yesavage, 1986).

### Statistical analysis

The group results are expressed as Median for all participants. We determined the *p* values for the measurement scores and biomarker concentrations using non-parametric statistics, setting the significance level to 0.05. We used the Wilcoxon signed rank test to examine the within-group effect and the Kruskal–Wallis test to examine the between-group effect using SPSS Windows software version 23.0.

## Results

A total of 28 participants’ Demian scores of MMSE, LOTCA, TIBSIT, GDS, and the levels of Tau and Tau and Aß1-42 at baseline are presented in Table 2. From the MMSE and LOTCA scores, all the participants had cognitive impairment. No significant differences in the MMSE, LOTCA, TIBSIT, and GDS scores were observed among the groups (*p* > 0.05) by using Kruskal–Wallis test, suggesting that cognitive impairment, olfactory function, and psychological status were identical at baseline. Because the *p* values for Tau and Aß1-42 were >0.05 by using Kruskal–Wallis test, no significant differences were observed among the groups at baseline.

**TABLE 2:** Non-parametric test result of pre and post-test for each group.

Test	OS (*N* = 9)				BG (*N* = 10)				CONT (*N* = 9)			
			
	Median		*Z*	*P*	Median		*Z*	*p*	Median		Z	*p*
							
	Pre	Post			Pre	Post			Pre	Post		
MMSE	17	18	−1.023	0.306	19	21.5	−1.440	0.150	15	17	−0.596	0.551
LOTCA	67	**71**	**−2.552**	**0.011**	71.5	76	−1.582	0.114	66	65	−1.011	0.312
TIBSIT	23	23	−0.772	0.440	23.5	21.5	−0.830	0.407	21	27	−1.198	0.231
GDS	3	0	−1.897	0.058	2	0	−1.725	0.084	1	0	−1.611	0.107
Tau (pg/ml)	22.94	21.06	−0.533	0.594	23.36	24.93	−1.172	0.241	21.11	22.41	−1.718	0.086
Aβ_1–42_ (pg/ml)	16.53	16.41	−1.008	0.314	16.11	16.53	−1.172	0.241	**15.48**	**16.77**	**−2.192**	**0.028**

OS, olfactory stimulation with cognitive training; BG, board game; CONT, control; MMSE, Mini-Mental State Examination; LOTCA, Loewenstein Occupational Therapy Cognitive Assessment; TIBSIT, Top International Biotech Smell Identification Test; GDS, Geriatric Depression Scale. The bold values mean statistical significant difference.

The MMSE, LOTCA, TIBSIT, and GDS median scores after the intervention, including the plasma Tau and Aß1-42 levels in the three groups, are also presented in Table 2. No significant differences in the post-test score of MMSE, LOTCA, TIBSIT, and GDS scores were observed among the groups (*p* > 0.05) using the Kruskal–Wallis test. The CONT group showed 22.41 pg/ml for the plasma Tau level and 15.48 pg/ml for the plasma Aß1-42 level. The OS group showed 21.06 and 16.41 pg/ml for the plasma Tau and Aß1-42 levels, respectively. Further, the BG group showed 24.93 and 16.53 pg/ml for the plasma Tau and Aß1-42 levels, respectively. Although there are no significant differences between the groups (*p* > 0.05) by using the Kruskal–Wallis test, the Tau level of the CONT group might be higher than the OS and BG groups (*p* = 0.054) after the intervention.

Using the Wilcoxon signed rank test analysis, we compared the pre and post-test scores of MMSE, LOTCA, TIBSIT, GDS, plasma Tau, and plasma Aß1-42 levels within three groups, as presented in Table 2. However, no significant changes were observed for the BG group. The OS group revealed an effective, increasing score of LOTCA after the intervention (*Z* = −2.552, *p* < 0.05), and the CONT group showed a significantly increased level of plasma Aß1-42 in the post-test (*Z* = −2.192, *p* < 0.05). These results may indicate that, following intervention, cognitive function significantly increased in the OS group, and biomarker concentration increased in the CONT group indicating worsen progression.

The aforementioned analysis is for between-groups; however, the intervention responses might be dependent on individual participants. The changes in plasma biomarkers after intervention should be analyzed for each participant using the concentration ratio expressed as ratio = after intervention concentration/baseline concentration. Because the ratio was found to be <1, the concentration was reduced after intervention compared with the baseline conditions. Correspondingly, the risks of neuronal damage or death (Tau) or cognitive decline (Aß1-42) are reduced.

**FIGURE 2:**
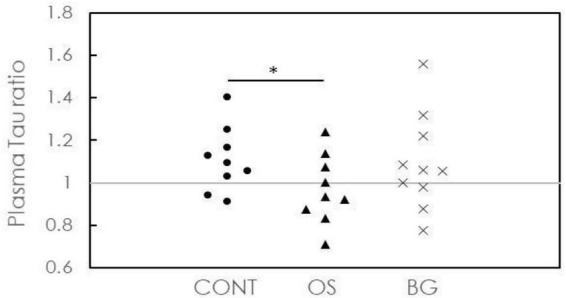
Concentration ratios of plasma Tau after the intervention to baseline for individuals in control (CONT), olfactory stimulation with cognitive training (OS), and board game (BG) groups. Each symbol means each subject’s concentration ratio of the plasma.

The ratios for plasma Tau in all groups are plotted in Figure 2. Two of 9 participants (22.2%) in the CONT group, 6 of 9 participants (66.7%) in the OS group, and 4 of 10 participants (40%) in the BG group showed plasma Tau ratios <1. Furthermore, the ratios in the OS group were significantly lower than those in the CONT and BG groups. However, we found no significant differences between these groups. These results indicate the feasibility of delaying neuronal damage or death in individuals with dementia by applying olfactory stimulation with cognitive training.

**FIGURE 3:**
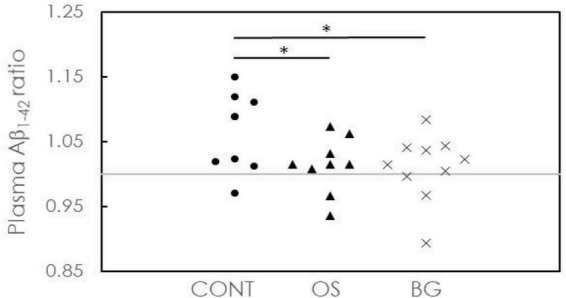
Concentration ratios of plasma Aß1-42 after intervention to baseline for individuals in the control (CONT), olfactory stimulation with cognitive training (OS), and board game (BG) groups. Each symbol means each subject’s concentration ratio of the plasma.

In Figure 3, the ratios for plasma Aß1-42 in all groups are plotted. One of 9 participants (11.1%) in the CONT group, 2 of 9 participants (22.2%) in the OS group, and 3 of 10 participants (30%) in the BG group showed ratios <1. More participants in the OS and BG groups showed a delay in cognitive decline than in the CONT group. As indicated by the distribution of the ratios in the OS and BG groups at ∼1, the biomarker level was nearly unchanged after intervention. However, the ratios in the CONT group were distributed in the region > 1, suggesting that the biomarker level was elevated following intervention. The *P* values between the OS and CONT groups (*p* = 0.028) and between the BG and CONT groups (*p* = 0.026) were >0.05. These results suggest that both OS and BG interventions could delay cognitive decline in individuals with dementia.

**FIGURE 4:**
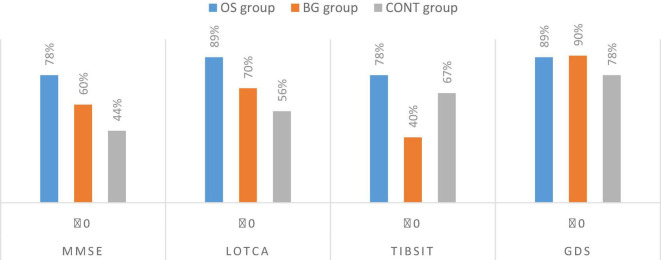
Shares of participants with stable or improved (≥0) cognition, olfactory and emotion.

As presented in Figure 4, 4 of 9 participants (44%) in the CONT group, 7 of 9 participants (78%) in the OS group, and 6 of 10 participants (60%) in the BG group had unchanged or higher MMSE scores after intervention. Five of 9 participants (55.6%) in the CONT group, 8 of 9 participants (89%) in the OS group, and 7 of 10 participants (70%) in the BG group had unchanged or higher LOTCA scores after intervention. Therefore, the cognitive decline in a higher number of participants stopped or was converted after the olfactory-based sensory stimulation or board game interventions. Six of 9 participants (67%) in the CONT group, 7 of 9 participants (78%) in the OS group, and 4 of 10 participants (40%) in the BG group had unchanged or higher TIBSIT scores after intervention. These results suggested that a higher number of participants performed better in the olfactory test after the olfactory-based sensory stimulation than in the absence of such stimulation. Although all groups showed similar shares of participants maintaining a stable or improving their psychological status, 7 of 9 participants (89%) in the OS group and 9 of 10 participants (90%) in the BG group showed a better emotional status after intervention.

## Discussion

Although no significant differences were observed in the three groups after the intervention, the OS group showed significant cognitive improvement in the LOTCA test. Further, the CONT group showed poor progression in plasma Aß1-42 levels. As can be seen from Table 2, the baseline plasma Aß1-42 level in the CONT group was 15.48 pg/ml, whereas the post-intervention level was 16.77 pg/ml (*P* = 0.011). Previous studies have detected increases in plasma Aß1-42 levels during clinical cognitive decline using the IMR technique (Lue et al., 2019). An elevation in plasma Aß1-42 levels as measured by IMR indicates an increased risk of cognitive impairment or Alzheimer’s disease, frontotemporal dementia, and vascular dementia (Chiu et al., 2012, 2020; Tang et al., 2018). In our study, the CONT group only performed daily activities that did not include experiencing intense olfactory stimulation or playing a cognitive game. The significant increase in plasma Aß1-42 levels in the CONT group after intervention was mainly due to the natural cognitive decline in neurodegenerative disorders (NDDs). Once participants were exposed to interventions such as olfactory stimulation and board games, their plasma Aß1-42 levels were nearly unchanged, suggesting that olfactory stimulation and board games could inhibit decline in NDDs.

The plasma Tau level in the CONT group at baseline was 21.11 pg/ml and slightly increased to 22.41 pg/ml after intervention, although this increase was not statistically significant. We also observed a similar increase in the BG group. However, the plasma Tau level decreased from 22.94 to 21.06 pg/ml after intervention in the OS group, although this increase was not statistically significant. Since elevation in the plasma Tau levels in individuals with dementia is mainly caused by neuronal damage or death, these results indicate that interventions such as olfactory stimulation with cognitive training can delay such damage or death.

Moreover, there was a higher share of participants in the OS group and BG group with stable or improved performance in cognition and emotion and with lower concentration ratios of plasma Tau and Aß1-42 than in the CONT group. Unfortunately, we could not find significant changes within the BG group. A systematic review and meta-analysis (Chen et al., 2022) verified that the board game (e.g., Mahjong, Go game, chess, and crossword puzzle) as an intervention could benefit global cognition and executive function in healthy older adults. However, few studies showed the effect on mild cognitive impairment or mild dementia. Their results revealed that the board game with more complex game rules, such as Mahjong, may trigger more human interaction and memory access for cognitive development. However, our study participants with a more severe degree of dementia and who could not play the game with too complex rules may have resulted in the consequence. Thus, olfactory stimulation with cognitive training is generally more effective than board games in maintaining stable cognition or inducing conversion in individuals with dementia and can elevate olfactory function and improve mood. Previous studies have confirmed the association between smell dysfunction and NDDs, particularly Alzheimer’s disease, frontotemporal dementia, and Parkinson’s disease (Sun et al., 2012). Furthermore, smell disturbance is correlated with psychiatric disorders, such as schizophrenia, depression, and bipolar disorder (Carnemolla et al., 2020; Ding et al., 2020). Although most studies have previously focused on developing an olfactory discrimination and identification test as a prescreening tool for early-onset dementia, our study indicated that olfactory stimulation with cognitive training could potentially improve cognitive function and depressive symptoms among individuals with mild-to-moderate dementia.

In addition to participating in olfactory-based sensory stimulation as the core intervention, the OS group also acquired several cognitive training skills after being asked to recognize, identify, remember, describe, and tell a story about the smell. Baker et al. (2001, 2003) found that multisensory stimulation and related activities had immediate effects on emotion and behavior initiation. Participants in their multisensory stimulation group were happier, more active or alert, and more initiative in attendance. However, an interactive activity such as playing a game or doing a puzzle with a clear goal and under the directive approach of a facilitator can yield greater benefits on mood and behavior. Maseda et al. (2014) revealed that the activity group using a directive approach and the multisensory stimulation environment group using a non-directive method might have a similar positive effect on the neuropsychiatric symptoms but not on the cognition for moderate to severe dementia. Without the cognitive component in the intervention, Jimbo et al. (2009) adopted aromatherapy but found no significant effect on cognition and emotion for people with dementia. In our study, the olfactory-based sensory stimulation intervention involved sensory stimulation and game activities in small groups. These results might indicate that sensory stimulation should not merely be a passive activity but be combined with directed activities to obtain more powerful results in the maintenance or conversion of cognitive decline.

One limitation in our study is the small number of participants. The calculated cut-off values, sensitivities, specificities, and AUCs would likely be modified as the case numbers increase. However, even with limited case numbers, significant differences in the plasma Tau ratio were observed between the OS and CONT groups, and in the plasma Aß1-42 ratio between the OS and CONT groups and the BG and CONT groups. The AUCs to discriminate OS or BG from CONT in terms of the plasma Tau or Aß1-42 ratios were higher than 70%, which would be expected to become higher as more subjects are enrolled. Another limitation was the impossibility to precisely diagnose the type of dementia based on the patients’ clinical diagnosis. Thus we adopted the biomarker test and multiple measurements to find more solid evidence. However, the participants in different groups are in the same unit, and the result might be interfered by each other. The concern is eliminated intentionally by comparing the effects with two comparisons. Furthermore, the intervention is short (30 min) and intensive (twice a week) because all the participants still regularly attended their routine activities. Future studies may suggest randomizing the study site to minimize the potential bias. Since we did not find any harmful or uncomfortable responses from the participants, the olfactory stimulation combined with cognitive training might be applied in the clinical setting. Further studies with a longer duration of intervention and follow-up examination are warranted to reveal cognitive change further. However, our preliminary results show promising effects on the use of olfactory stimulation and board games to stabilize or delay cognitive decline in individuals with dementia. Multiple sensory stimulation during activities such as therapeutic horticulture could be developed into treatment programs to be regularly delivered in daycare centers for older people and to explore the long-term effect on the broader perspective, such as quality of life.

## Conclusion

Among individuals with dementia who received olfactory-based sensory stimulation or board game interventions, >50% of participants were found to have stable or improved cognitive impairment. Plasma Tau and Aß1-42 levels were well controlled in these participants. To the best of our knowledge, this is the first study to investigate the effects of such interventions on cognitive abilities using plasma Aß1-42 and Tau levels. We conclude that olfactory stimulation with cognitive training is more effective than board games. Larger cohort studies are warranted to validate these findings further.

## Data availability statement

The original contributions presented in this study are included in the article/supplementary material, further inquiries can be directed to the corresponding author.

## Ethics statement

The studies involving human participants were reviewed and approved by Research Ethics Committee at the National Taiwan Normal University (NTNU-REC 201901HM030). The patients/participants provided their written informed consent to participate in this study.

## Author contributions

L-JL planned the study, performed all statistical analyses, and wrote the manuscript. K-YL helped to plan the study, including the instrumentation, and to revise the manuscript. Both authors contributed to the article and approved the submitted version.
